# Contents of Visual Predictions Oscillate at Alpha Frequencies

**DOI:** 10.1523/JNEUROSCI.0474-25.2025

**Published:** 2025-10-16

**Authors:** Dorottya Hetenyi, Joost Haarsma, Peter Kok

**Affiliations:** ^1^Department of Imaging Neuroscience, UCL Queen Square Institute of Neurology, University College London, London WC1N 3AR, United Kingdom; ^2^Department of Cognitive Neuroscience, Faculty Psychology and Neuroscience, Maastricht University, Maastricht 6229 EV, The Netherlands

**Keywords:** expectation, perceptual inference, pre-stimulus oscillations, predictive processing, stimulus templates, time-resolved multivariate analysis

## Abstract

Predictions of future events have a major impact on how we process sensory signals. However, it remains unclear how the brain keeps predictions online in anticipation of future inputs. Here, we combined magnetoencephalography (MEG) and multivariate decoding techniques to investigate the content of perceptual predictions and their frequency characteristics. Thirty-two participants (23 female) were engaged in a shape discrimination task, while auditory cues predicted which specific shape would likely appear. Frequency analysis revealed significant oscillatory fluctuations of predicted shape representations in the pre-stimulus window in the alpha band (10–11 Hz). Furthermore, we found that this stimulus-specific alpha power was linked to expectation effects on shape discrimination behavior. Our findings demonstrate that sensory predictions are embedded in pre-stimulus alpha oscillations and modulate subsequent perceptual performance, providing a neural mechanism through which the brain deploys perceptual predictions.

## Significance Statement

Prior knowledge greatly influences how we perceive the world. However, it is unclear how the brain maintains and keeps prediction signals in the anticipation of future inputs. Our study reveals a neural mechanism by which the brain maintains sensory predictions. We demonstrated that perceptual predictions are encoded in pre-stimulus alpha oscillations (10–11 Hz). These oscillations not only reflect the content of predicted visual shapes but are also directly linked to enhanced perceptual performance. These findings provide key insights into how the brain prepares for and improves perception of future sensory inputs, contributing to our understanding of predictive processing in human cognition.

## Introduction

Predictions about how the world is structured play an integral role in perception (Friston, 2009; Bastos et al., 2012; Clark, 2013; de Lange et al., 2018). Our prior knowledge forms the basis for predictions of future sensory events, which are subsequently integrated with sensory input to form a perceptual experience. While there is a wealth of evidence supporting the idea that the brain deploys predictions to guide perception, the mechanisms through which the brain keeps these predictions online remain largely unknown. One likely candidate for conveying perceptual predictions are neural oscillations (Arnal and Giraud, 2012; Mayer et al., 2016; Auksztulewicz et al., 2017; Bastos et al., 2020).

Alpha rhythms (8–12 Hz) are the predominant oscillations in the awake human brain (Berger, 1929), yet their functional role is controversial (Klimesch et al., 2007; Jensen and Mazaheri, 2010). The amplitude and phase of these ongoing oscillations is known to influence performance in visual tasks (Ergenoglu et al., 2004; van Dijk et al., 2008; Busch et al., 2009; Mathewson et al., 2009; Romei et al., 2010; Dugué et al., 2011; VanRullen et al., 2011; Hanslmayr et al., 2013; Iemi et al., 2019) and have been found to vary with experimental manipulations that target stimulus predictability (Sherman et al., 2016; Alilović et al., 2019). Specifically, pre-stimulus alpha oscillations have a similar topography to post-stimulus responses, implying a shared neural substrate in the processing of pre-existing information and external stimuli (Shen et al., 2023), alpha phase determines the influence of predictions on perception (Sherman et al., 2016), and alpha power has been shown to predictively encode the position of a moving stimulus (Turner et al., 2023). Furthermore, alpha-band activity modulates sensory processing of both low-level features and high-level object representations through selective attention, suggesting a hierarchical and predictive role in top-down control (Noah et al., 2020; Meyyappan et al., 2024). However, whether these oscillations actually convey the contents of perceptual predictions remains unknown.

To test this hypothesis, we combined magnetoencephalography (MEG) with multivariate decoding analyses to resolve visual representations with millisecond resolution and characterize the temporal and frequency characteristics (Kerrén et al., 2018, 2022) of sensory predictions (Stecher and Kaiser, 2024; Stecher et al., 2025). Participants were engaged in a shape discrimination task where auditory cues predicted the identity of upcoming abstract shapes. We identified the neural representations of cued sensory predictions prior to stimulus onset and tested whether these sensory predictions had an oscillatory nature, as well as whether the power of such predictive oscillations modulated perceptual performance (Tarasi et al., 2022).

## Materials and Methods

### Participants

Sixty-two healthy right-handed participants (43 female) with normal or corrected-to-normal vision and no history of neurological disorders took part in the behavioral experiment. This experiment served as a pre-assessment process to familiarize the participants with the task and select only those whose average performance accuracy on the challenging shape discrimination task was above 70% across the four runs. Thirty-nine participants (28 female) met the performance inclusion criteria and participated in the MEG experiment. Seven participants were excluded from subsequent analyses due to excessive head movement (*N* = 5) or not completing the full experiment (*N* = 2), leaving 32 participants (23 female, age 26 ± 5 years, mean ± SD) for the MEG analysis.

### Stimuli

**Figure 1.**
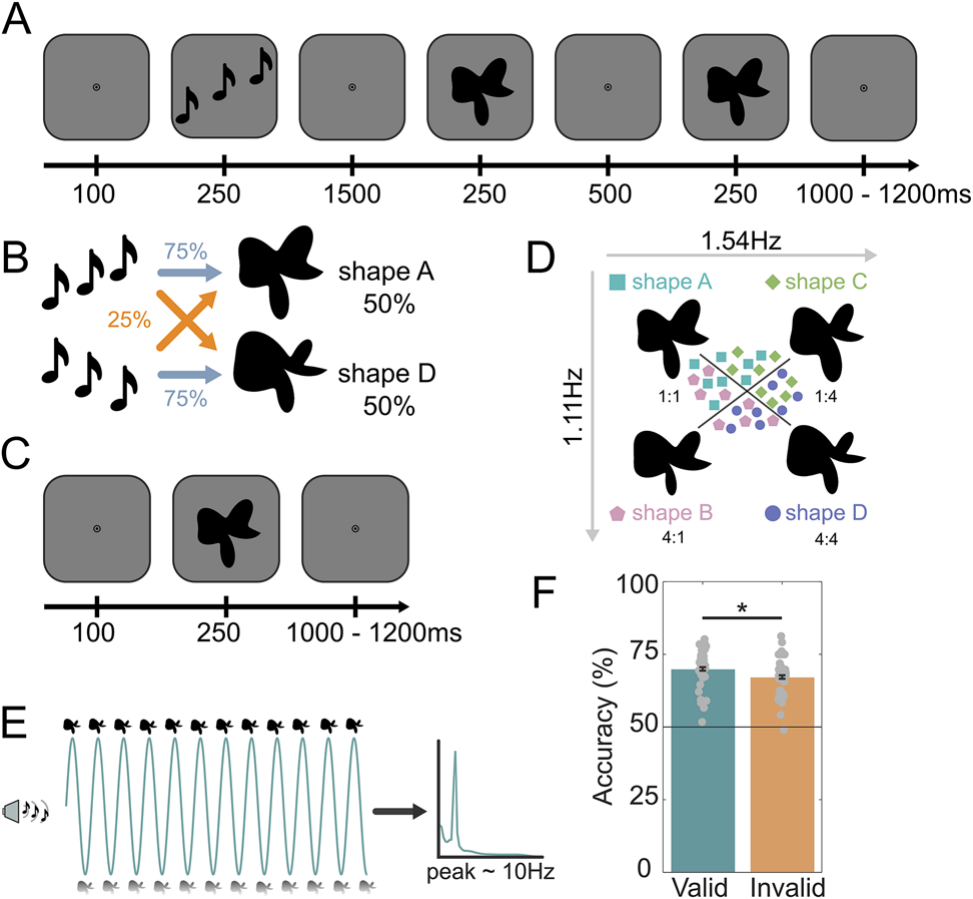
Experimental paradigm. ***A***, During prediction runs, an auditory cue preceded the presentation of two consecutive shape stimuli. On each trial, the second shape was either identical to the first or slightly warped with respect to the first, and participants' task was to report whether the two shapes were the same or different. ***B***, The auditory cue (rising vs falling tones) predicted whether the first shape on that trial would be shape A or shape D. The cue was valid on 75% of trials, whereas in the other 25% of (invalid) trials the unpredicted shape was presented. ***C***, During shape localizer runs, no predictive auditory cues were presented and participants performed a fixation diming task. ***D***, Four different shapes were presented in the localizer runs, appearing with equal (25%) likelihood. Only shape A and D were presented in the prediction runs. The amplitudes of two RFCs (1.11 and 1.54 Hz components) were varied in order to create a two-dimensional shape space multiplication indicated shape A (1:1); shape B (4:1); shape C (1:4); shape D (4:4), such that shape A versus D discrimination was orthogonal to shape B versus C discrimination. ***E***, Schematic of the hypothesis: cue-induced predictions oscillate in the alpha frequency band (∼10 Hz) in the interval between predictive cue and stimulus onset. ***F***, Participants were able to discriminate the two presented shapes more accurately when the auditory cue validly predicted the identity of the first shape (*p < 0.05). Dots represent individual participants; error bars indicate within-participant SEM (Cousineau, 2005; Morey, 2008).

The experiment employed the same design as Kok and Turk-Browne (2018), wherein participants discriminated between two consecutively presented shapes which were preceded by a predictive auditory cue. Each predictive cue was composed of three pure tones (440, 554, and 659 Hz; 80 ms per tones; 5 ms intervals), played with rising or falling pitch, with a total duration of 250 ms. Visual stimuli were generated using MATLAB (The MathWorks, version 2021b) and Psychophysics Toolbox (Brainard, 1997). The visual stimuli consisted of complex abstract shapes defined by radial frequency components (RFCs), a method where shape contours are created by modulating the radius of a circle as a function of angular position (Zahn and Roskies, 1972). This technique allows for precise control of shape complexity and variation by adjusting the frequency and amplitude of sinusoidal modulations around the shape's circumference. To generate the contours of the stimuli, seven RFCs (0.55, 1.11, 4.94, 3.39, 1.54, 3.18, 0.57 Hz) were used which were based on a subset of stimuli from Op de Beeck et al.'s work [Op de Beeck et al. (2001), their Fig. 1A]. To construct a two-dimensional shape space, we systematically varied the amplitudes of two RFCs (specifically the 1.11 and 1.54 Hz components) while keeping the amplitudes of the other components constant (indicated by the arrows in Fig. 1*D*). These variations created the four distinct abstract shapes such as the following: shape A (baseline amplitudes, amplitude of 1 × 1.11 Hz = 8; 1 × 1.54 Hz = 8); shape B (amplitude of 1 × 1.11 Hz = 8; 4 × 1.54 Hz = 26); shape C (amplitude of 4 × 1.11 Hz = 26; 1 × 1.54 Hz = 8), and shape D (amplitude of 4 × 1.11 Hz = 26; 4 × 1.54 Hz = 26). These shapes were designed such that discrimination between shapes A and D (both amplitudes changed) was orthogonal to the discrimination between shapes B and C (only one amplitude varied), defining a clear two-dimensional shape space (Fig. 1*D*). As explained above, the shapes were designed in a very precise and controlled way, accounting for discrimination accuracy perceptually and neuronally. This allowed us to study signals reflecting complex visual shapes, rather than conventional gratings, yet in an experimentally controlled manner. Additionally, RFC-based warping was used to generate moderately distorted versions of the two main experiment shapes (Fig. 1*D*, shapes A and D) for the benefit of the shape discrimination task. This warp to define the shape was achieved by modulating a different RFC's amplitude (3.18 Hz) than the two used (1.11 and 1.54 Hz) to define the shape space. This modulation could be either positive or negative (counterbalanced over conditions) and was orthogonal to the shape space used for the two main experiment shapes and therefore to the cue predictions as well. The visual stimuli were displayed on a rear-projection screen using a projector (1,024 × 768 resolution, 60 Hz refresh rate) against a uniform gray background.

### Behavioral experiment

The study had two parts, a behavioral training and screening experiment, and an MEG experiment for those who passed the behavioral screening. In both parts, participants were engaged in a shape discrimination task. Each trial started with a fixation bullseye (diameter, 0.7°) for 100 ms, followed by the presentation of two consecutive shape stimuli each for 250 ms, and separated by a 500 ms blank screen containing only a fixation bullseye (Fig. 1*A*). On each trial, the second shape was the same as the first or slightly warped. The modulation was either positive or negative, and the size of the modulation was determined by an adaptive staircasing procedure (Watson and Pelli, 1983), updated after each trial, in order to make the task challenging. Participants were instructed to report whether the two presented shapes were identical or different. After the response interval ended (750 ms after disappearance of the second shape), the fixation bullseye was replaced by a single dot, signaling the end of the trial while still prompting participants to fixate. On each trial, one of the four shapes (Fig. 1*D*, shape A, B, C, or D) was presented, in a counterbalanced (i.e., non-predictable) manner. Participants performed four runs (360 trials in total) of the shape discrimination task, maximum 1 week prior to the MEG session.

### MEG experiment

The MEG experiment started with two localizer runs, containing the same four abstract shapes as in the behavioral task. To ensure participants were engaged, they performed a fixation dimming task (10% of total trials, ∼24 of 248 trials per run). Each trial began with a fixation bullseye (visual angle: 0.7°) displayed for 100 ms, followed by one of the four shapes presentation for 250 ms. Following the stimulus presentation, the fixation bullseye reappeared and remained on the screen for a period between 1,000 and 1,200 ms. In 10% of the trials, fixation bullseye dimmed for 150 ms, and participants had been instructed to press a button when this occurred. By using identical stimulus durations, these runs were designed to be as similar as possible in terms of stimulus presentation to the main experiment. During the localizers, participants correctly detected 95.3 ± 0.7% (mean ± SEM) of fixation dimming events and incorrectly pressed the button on 4.9 ± 2.2% of trials, suggesting that participants were successfully engaged by the fixation task.

Following the localizer runs, participants performed 8 main task runs (2× training runs, 6× prediction runs), 64 trials per run, in total 512 trials. During the prediction runs, an auditory cue (falling vs rising tones, 250 ms) was presented 100 ms after trial onset. Following a 1,500 ms interval, two consecutive shape stimuli were displayed (each for 250 ms) and separated by a 500 ms blank screen (Fig. 1*A*). As in the behavioral session, participants' task was to indicate whether the two shapes were the same or different. The auditory cue predicted whether the first shape presented on that trial would be shape A or D. The cue was valid on 75% of trials, whereas on the other 25% of trials the unpredicted shape would be presented (Fig. 1*B*). For instance, if the cue was a falling auditory tone, it might lead to shape A in 75% of cases and shape D in the other 25% of cases. The prediction induced by the auditory cue (predicting the identity of the first shape) was thus orthogonal to participants' task (whether the two shapes were the same or different). This experimental design choice was made to prevent the cues from inducing responses biases in the task (cf. Kok et al., 2012a, 2017; Kok and Turk-Browne, 2018). Despite this orthogonality, previous work using a similar design has revealed a significant benefit in behavioral performance of valid prediction cues, which was correlated with neural effects of the predictive cues (Kok et al., 2012a, 2017; Kok and Turk-Browne, 2018). Additionally, ensuring the predictive cues were orthogonal to the task allowed us to manipulate expectation while keeping attention constant, since both validly and invalidly cues shapes were equally task relevant.

Note that shapes B and C were never presented in the prediction runs. The contingencies between cues and shapes were flipped halfway through the experiment, and the order was counterbalanced over subjects. Prior to the first prediction run, and after the cue reversal halfway through, participants were trained on the cue–shape associations during training runs in the MEG and explicitly informed about the cue contingencies. In the training runs, the auditory cue was 100% predictive of the identity of the first shape.

### Preprocessing

Whole-head neural recordings were obtained using a 273-channel MEG system with axial gradiometers (CTF Systems) at a rate of 600 Hz located in a magnetically shielded room. Throughout the experiment, head position was monitored online and corrected if necessary using three fiducial coils that were placed on the nasion, right and left preauricular. If participants moved their head >5 mm from the starting position, they were repositioned after each run. Eye movements were recorded using an EyeLink 1000 infrared tracker (SR Research). The recorded eye-tracker data were used to identify eyeblink-related artifacts in the MEG signal. Auditory tones were delivered using earplugs (Etymotic Research). A photodiode was placed at the bottom left corner of the screen to measure visual stimulus presentation latencies. The photodiode signal was used to realign the MEG signal with stimulus onset.

The data were preprocessed offline using FieldTrip (Oostenveld et al., 2011). The variance (collapsed over channels and time) was calculated for each trial in order to identify artifacts. Trials with large variances were subsequently selected for manual inspection and removed if they contained excessive and irregular artifacts. Average number of trials removed per participants was 5.74 ± 4.45% for valid condition, 5.76 ± 5.47% for invalid condition, 5.66 ± 4.43% for presented shape A and 5.83 ± 5.01% for presented shape D (mean ± SD). Next, independent component analysis was used to further remove cardiac and eye movement-related artifacts. The independent components were correlated to the eye tracking signal to identify potentially contaminating components for each participant and inspected manually before removal. Notch filters were also applied at 50, 100, and 150 Hz to remove line noise and its harmonics. No detrending was applied for any analysis. Finally, main task data were baseline corrected on the interval of −200 to 0 ms relative to auditory cue onset, and localizer data were baseline corrected on the interval of −200 to 0 ms relative to shape onset.

### Decoding analysis

To uncover the representational content of neural activity, we performed a decoding analysis using a customized linear discriminant analysis (LDA) decoder. LDA aims to find a linear transformation of the data, where the resulting signal is optimally discriminative between two labels. The data fed into the decoder was separated based on the number of MEG channels (**F**) at each time point; therefore, $\hat{\mu_{1}}$ and $\hat{\mu_{2}}$ were column vectors of length **F** that contain the neural responses in the training set for labels 1 and 2. The weights vector (**w**) that optimally discriminates between labels on the basis of the channels was calculated as $w\;=\;{\tilde{\Sigma }}_{C}^{-1}(\hat{\mu_{2}}-\hat{\mu_{1}})$. ${\tilde{\Sigma }}_{C}$ is the common regularized covariance matrix. The data to be decoded (**X**) was set as a matrix of size channels X trials (**N**), and the decoded data (**y**) was then obtained, where **T** denotes the matrix transpose as $y\;=\;w^{T}X$. Rather than assigning discrete labels, the decoder outputs a continuous measure of label encoding strength in the signal for each single trial. Therefore, no binary cutoff was applied to the decoded data. Retaining single trial data allowed us to perform across-trial analysis (such as coherence and logistic regression against behavioral reports, see below). A normalization factor was applied to allow comparing the data across time points (i.e., the mean difference in the decoded data between labels was equal to a value of one). The equation describing the decoder, including the normalization factor, as described in Mostert et al. (2015), was as follows:

$$w\;=\;\frac{{\hat{\Sigma }}_{C}^{-1}\;(\hat{\mu_{2}}-\hat{\;\mu_{1}})}{{\left(\hat{\mu_{2}}-\hat{\;\mu_{1}}\right)}^{T}\;{\hat{\Sigma }}_{C}^{-1}(\hat{\mu_{2}}-\hat{\;\mu_{1}})}.$$

This decoder assessed how sensor-level activity varied based on a discriminability index, providing a continuous measure of shape decoding on a trial-by-trial basis. The decoding analysis was time resolved. First, the data were downsampled (from the original sampling rate of 600 Hz to 200 Hz) using a 28 ms sliding time window centered on each time point, with the window advancing in 5 ms steps. Then, the decoder was applied to the downsampled data, resulting in a decoder output matrix of trials × training time × testing time.

To test how effective the decoder was at revealing neural patterns, it was first trained and tested on shape A and D trials (between −100 and 600 ms relative to stimulus onset) from the localizer runs, using a leave-one-block-out approach. Analogously, a shape B versus C decoder was tested on shape B and C trials (Fig. S1*D*). To further validate the analysis, we tested the shape A versus D decoder on the shape B and C trials, and the shape B versus C decoder on shape A and D trials (Fig. S1*B,C*). We expected significant decoding within shape categories (e.g., training and testing on shape A vs D), but not across shape categories (i.e., training on shape A and D and testing on shape B and C, and vice versa).

Localizer decoding results were analyzed using nonparametric cluster-based permutation tests. The data were represented as 2D matrices of decoding performance, with training time on one axis and testing time on the other. The statistical analysis focused on identifying significant 2D clusters in these matrices. To do so, univariate *t* statistics were calculated for the entire matrix. Elements that were considered neighbors, i.e., directly adjacent in cardinal or diagonal directions, were collected into separate positive and negative clusters if they passed a threshold corresponding to a *p* value of 0.001 (two-tailed). The significance of the clusters was assessed by summing the *t* values within each cluster to obtain cluster-level test statistics. These test statistics were then compared with a null distribution, which was created by randomly shuffling the observed data 10,000 times. A cluster was considered significant if its resulting *p* value was <0.05 (two-tailed).

**Figure 2.**
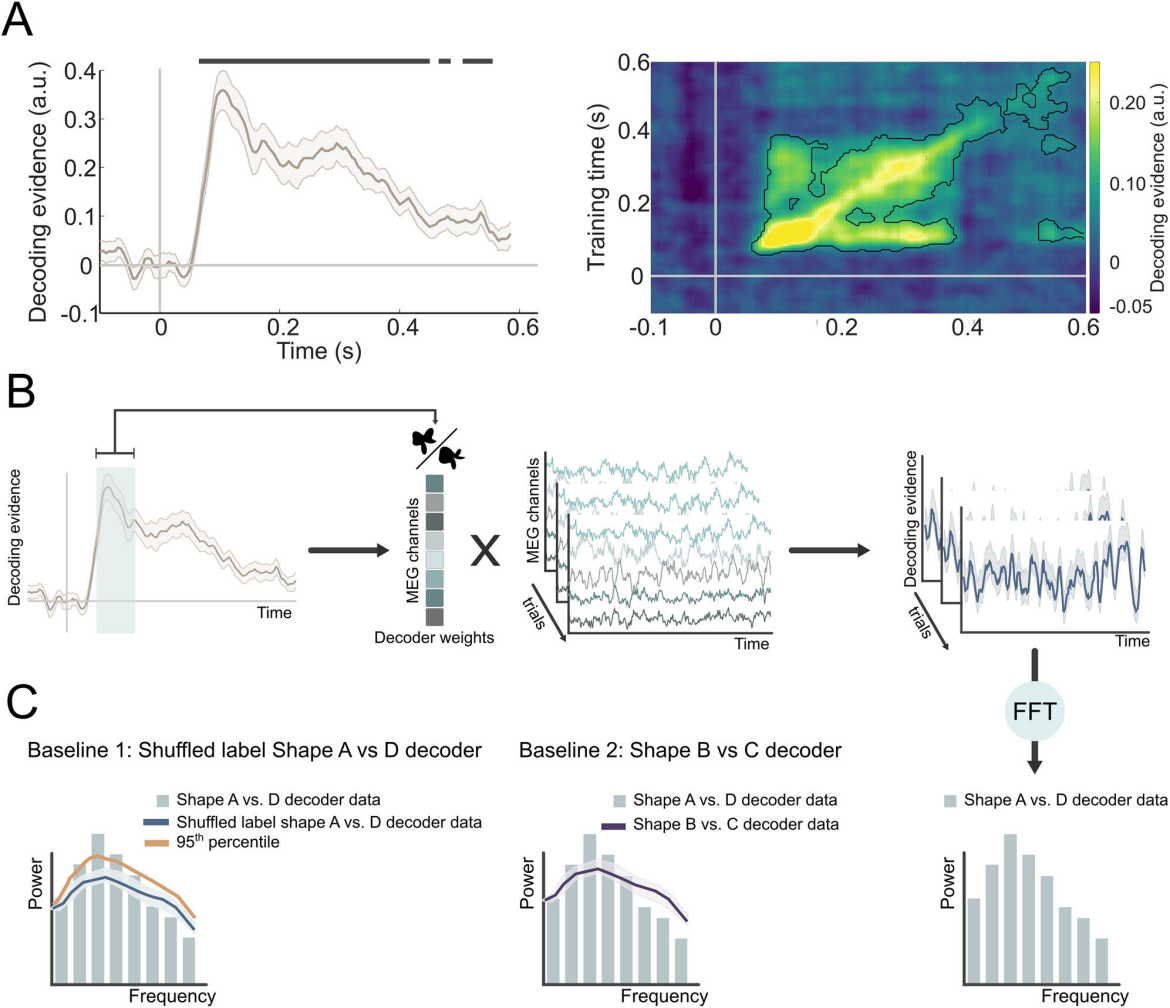
Shape prediction frequency analysis pipeline. ***A***, Neural representations of the presented shapes within the localizer runs in a cross-validated manner (−100 to 600 ms, relative to stimulus onset). The decoder was highly accurate at discriminating the shapes based on the MEG signal. The presented shapes were successfully decoded from 65 to 450 ms (*p* < 0.001). For all subsequent analyses, decoding traces were averaged over a training window of 70–200 ms, during which shape decoding peaked. ***B***, A decoder was trained to discriminate between shapes A and D in the localizer runs. This decoder was applied to the pre-stimulus time window in prediction runs (−1,250 to 0 ms). Trial-based pre-stimulus decoding time series were subjected to FFT. ***C***, The resulting power spectrum was compared with the 95th percentile of an empirical null distribution generated by bootstrapping decoders trained with pseudorandomized labels (Baseline 1, left), as well as to a decoder trained on shapes only presented in the localizer (shapes B and C; Baseline 2, right).

In order to reveal predicted shape representations, the decoder was trained on shape A versus D localizer trials (70–200 ms) again in a time-resolved manner with the identical parameters as above and subsequently tested on the pre-stimulus window (−1,750 to 0 ms relative to shape onset) during the prediction runs (Fig. S2). To address label imbalances resulting from trial rejections during preprocessing, random resampling was applied to the training sets, ensuring an equal number of each decoded classes (shapes) for every participant. Furthermore, we repeated the same procedure for each participant using a control decoder trained on shapes B versus C localizer trials, i.e., shapes which were not presented during the prediction runs. These results of applying this control decoder to the pre-stimulus prediction window served as a baseline (Fig. 2*C*, right, Baseline 2) in further analyses. It is important to highlight that the shape B versus C discrimination was orthogonal to shape A versus D discrimination.

### Frequency analysis of pre-stimulus decoding time series

Our primary aim was to test whether the decoded neural representations of predicted shapes had oscillatory dynamics. Therefore, we adapted the analysis approach of Kerrén et al. (2018), investigating the frequency characteristics of decoder time series using FFT. This analysis examined whether the spatial shape-specific pattern identified by training an LDA decoder on shape-specific evoked signals (in the localizer runs) showed evidence of rhythmic fluctuations during the cue–stimulus prediction interval. The LDA shape decoders were trained on the trial-by-trial evoked responses during the shape localizer in a time-resolved manner (sliding window of 28 ms, steps of 5 ms). These decoders were applied to the cue–stimulus prediction intervals (−1,250 ms to 0 ms relative to the onset of the first shape) in order to reveal shape-specific sensory prediction signals. Based on the results of cross-validated within-localizer shape decoding (Fig. S1*A*), we averaged over decoding training time points in the 70–200 ms post-stimulus window for subsequent analyses. The decoder output (as mentioned above) contained a continuous measure of shape discrimination, rather than binarized class information. Using decoding accuracy alone would not allow us to explore the oscillatory nature of the neural signal, as continuous decoder output reflects a nongraded shape information. Additionally, trial-by-trial phase misalignment may impose a challenge, as we did not expect the predictive signal to occur at the exact same latency across participants, averaging across trials to compute accuracy could harm meaningful temporal dynamics. Keeping the continuous output better preserves this information for subsequent frequency analysis.

For each participant, each trial of the pre-stimulus decoded time series was tapered with a Hann window covering the whole time period (−1,250 to 0 ms) and then subjected to an FFT. In a control analysis, we used Fitting Oscillations and One-Over-F (FOOOF), as implemented in the FieldTrip toolbox (Donoghue et al., 2020), which separates rhythmic activity from concurrent power-spectral 1/*f* modulations in electrophysiological data, to validate the oscillatory nature of the predictive representations (Fig. S3*A*). We also repeated the frequency analysis in a time-resolved manner (between −2,000ms and 500 ms, with a fixed-length 500 ms Hann window running at every 50 ms) to be able to visualize the extent of the alpha oscillations (Fig. S3*C*).

To assess the reliability of our results, we created an empirical baseline using decoders with randomly shuffled shape labels (Fig. 2*C*, left, Baseline 1). The labels of the two shapes (shape A and D) were shuffled pseudorandomly before training the decoder, 25 times per participant. Therefore, each participant yielded 25 permuted datasets. The analysis parameters for the baseline decoding were identical to the nonshuffled decoder, i.e., identical spectral analysis was performed for each of the 25 datasets per participant. We generated an empirical null distribution by bootstrapping the permuted datasets (*n* = 10,000; Stelzer et al., 2013) and compared this to the frequency analysis results of the nonshuffled shape A versus D decoder data (Kerrén et al., 2018). Frequency bins with higher power than the empirical null distribution (exceeding the 95th percentile) were considered significant. To further validate the findings, we also conducted the identical frequency analysis (same analytical parameters) using decoders trained to distinguish shape B versus C rather than A versus D as an additional baseline (Fig. 2*C*, right, Baseline 2). Importantly, shapes B and C were highly similar to shapes A and D but lay on an orthogonal discrimination axis (Fig. 1*D*), yielding a very precise and stringent control analysis. To correct for multiple comparisons, we extracted the *p* values for each frequency bin (1–99 Hz) relative to the Baseline 1 data and applied the false discovery rate (FDR) correction. The *p* values were derived using a one-sided test versus Baseline 1, to test specifically whether the power in the shape A versus D decoder was higher than in the baseline. To test whether the alpha fluctuations observed in the decoder traces were indeed driven by alpha-band–specific MEG signals, we applied bandpass filtering using a one-pass Butterworth filter with a filter order of four between 8 and 12 Hz on the main task (prediction runs) sensor-level MEG data for all MEG channels. Then, the bandpass-filtered MEG time courses were subjected to the identical decoding and frequency analysis as described above (Fig. S3*B*).

In an exploratory analysis, we repeated the analysis for two shorter training time windows (90–120 ms and 160–190 ms), centered around the first (105 ms) and second peak (175 ms) of localizer shape decoding (Fig. 2*A*) to distinguish effects of earlier and later visual representations. These time windows were chosen since they appeared to form distinct clusters in the localizer decoding temporal generalization matrix, with reduced cross-decoding between the two clusters suggesting qualitatively different representations (Fig. S1*A*).

### Relating behavioral and neural effects of predictions

To investigate whether there was a relationship between shape-specific pre-stimulus alpha power and shape discrimination performance, we performed a logistic regression analysis separately for valid and invalid prediction trials. Based on the existing literature relating pre-stimulus oscillatory power and phase to behavioral performance (Hanslmayr et al., 2007; Mayer et al., 2016; Samaha et al., 2018), we limited the pre-stimulus decoding time series to −500 to 0 ms relative to stimulus onset. To be able to accurately estimate pre-stimulus alpha power, yet be as close as possible to stimulus onset, we used a fixed-length 500 ms Hann window over the −500 to 0 ms time window, resulting in ∼2 Hz frequency resolution (alpha frequency bins: 7.812, 9.375, 12.500 Hz). Separately for valid and invalid prediction trials, trial-based power estimates of the pre-stimulus (−500 to 0 ms) alpha activity were *z*-scored and averaged over for the three alpha frequency bins. Identical frequency analyses were applied to the raw MEG signal (−500 to 0 ms, averaged over occipital channels) to quantify trial-based raw alpha power. We balanced the trial numbers by randomly choosing (*n* = 1,000 times) a subset of trials from the conditions with higher trial counts (i.e., valid). The dependent variable of the model was the behavioral outcome (correct or incorrect response), sorted separately again for valid and invalid predictions. Independent variables were shape-specific alpha power and raw alpha power. Raw alpha power was added to the model to control for non-shape-specific alpha effects on behavior. The model parameter estimates (i.e., beta values) served as an indication of an underlying link between alpha power and behavioral performance. The valid and invalid condition beta values were statistically compared using a paired *t* test.

### Coherence between shape-specific pre-stimulus fluctuations and raw alpha oscillations

To further assess the relationship between shape-specific alpha fluctuations (i.e., fluctuations in the decoder time courses) and neural alpha oscillations, we estimated the coherence between the two signals on a trial-by-trial basis at the sensor level and in source space. The coherence value is a number between 0 and 1, which reflect the consistency of the phase difference between the two signals at a given frequency. Cross-spectral density between *z*-scored MEG signals of all MEG channel combinations and between the MEG channels and the *z*-scored decoder traces were calculated across trials at our frequency of interest (10 Hz with 2 Hz smoothing) between −1,250 and 0 ms. In order to localize the neuronal sources which were coherent with the decoder traces, we applied the DICS (Dynamic Imaging of Coherent Sources; Gross et al., 2001) beamformer, which is specifically designed to localize sources coherent with another time series signal. We applied a regularization parameter of lambda = 5% to decrease the effect of noise on the source estimates. To additionally visualize the source of raw alpha oscillations, we repeated the beamformer analysis on the frequency decomposed MEG data at 10 Hz with 2 Hz smoothing including all MEG channels. The spatial filter was calculated (i.e., “common filter”) on the MEG data containing both pre- (−1,250 to 0 ms) and post-stimulus (0 to 1,250 ms) and then applied in separately both time periods. The main advantage of using a common filter is that more data is used to construct the spatial filters (resulting in more reliable filters), and any difference in source activity can be attributed to power differences between the conditions, rather than to discrepancies between the filters. Based on previous studies demonstrating that the anatomical specificity gain of using subject-specific anatomical images is negligible (Holliday et al., 2003), we did not collect individual anatomical MRI scans for our subjects. We followed a group-based template approach using a template MRI (in MNI space) in combination with a single shell head model and a standard volumetric grid (8 mm resolution), as present in the FieldTrip toolbox. Participants' individual fiducials were used to generate a participant-specific forward model in MNI space by wrapping the template head and source model to the participants' fiducials. Then, these participant-specific head and source models were included in the beamforming analysis.

### Source localization of shape decoding

To visualize the underlying neural sources during decoding, we applied source localization analyses using an LCMV beamformer (Van Veen et al., 1997). The spatial distribution of the underlying signal during classification in LDA is primarily influenced by the magnetic field difference between the two experimental conditions. Therefore, one can visualize the source of a decoder by estimating the sources of the two different conditions and compute the difference (Haufe et al., 2014). The spatial filter was computed for the time window of interest (70–200 ms, i.e., decoder training window) in the averaged data, which was subsequently applied separately to the two conditions of interest (shape A and D trials). For shape A versus D decoding, a percentage absolute signal change was computed in source space, to determine which source signals were involved in discriminating between shape A and D without making assumptions about the sign of the dipole (Fig. S1*E*).

## Results

### Predictive cues lead to improved shape discrimination accuracy

We tested whether the predictive cues affected behavioral performance. As a reminder, participants were required to indicate whether two shapes presented in succession were the same or different. It should be noted that any effects of the predictive cues on performance are not trivial, given that the shape discrimination task was orthogonal to the prediction manipulation (i.e., the cues predicted the identity of the first shape, but did not inform participants whether the two shapes would be identical or different). Still, valid predictive cues might improve performance indirectly by enhancing processing of the initial shape, facilitating discrimination of the subsequent shape (Kok et al., 2012a, 2017). Vice versa, invalid cues might perturb performance by impeding the processing of the initial shape. In line with this, shape discrimination accuracy was significantly influenced by whether the auditory cue correctly predicted the identity of the first shape (accuracy valid = 70% ± 1.2% and accuracy invalid = 67% ± 1.3%, mean ± SEM; *t*_(31)_ = 3.215, *p* = 0.003; Fig. 1*F*). There was no difference in reaction times (valid = 614 ± 0.013 ms and invalid = 615 ± 0.013 ms, mean ± SEM; *p* = 0.626, *t*_(31)_ = −0.492). Together, this suggests that valid predictions facilitated shape processing, leading to improved discrimination performance.

### Predictions oscillate at alpha frequencies

To test whether perceptual predictions are conveyed by oscillations, 32 participants performed a challenging visual shape discrimination task, reporting whether two consecutive shapes were the same or different (Fig. 1*A*). On each trial, an auditory cue predicted the identity of the first shape (shape A or D, 75% valid; Fig. 1*B*). The shape discrimination task was orthogonal to the prediction manipulation, i.e., the auditory cue did not convey any information about whether the two shapes would be identical or different.

To reveal the representational content of neural activity, a decoding analysis was applied. We used an LDA decoder, which described how activity at the sensor-level varied as a function of a discriminability index. Unlike conventional LDA which separates data into discrete categories, our customized decoder calculated the distances of each test sample to the hyperplane, treating these distances as discriminant evidence (Mostert et al., 2015; Kok et al., 2017). Thereby, we obtained a continuous measure of which shape was encoded in the neural signals, providing finer resolution in analyzing the neural representations. For details on the implementation of the LDA, see methods (Mostert et al., 2015). The decoding analysis was performed in a time-resolved manner by applying it sequentially at every single time point on data prior downsampled (with a sliding time window of 28 ms in steps of 5 ms). First, we identified shape-specific neural signals by applying the LDA to MEG responses evoked by task-irrelevant shapes (70–200 ms post-stimulus) during separate shape localizer runs (Fig. 1*C*; Fig. S1*A*). Localizer runs consisted of the presentation of four abstract shapes (Fig. 1*D*), which were designed to lie on two orthogonal axes of perceptual and neural discriminability, allowing us to train a decoder that was sensitive to predicted shape information (shape A vs D) as well as a baseline decoder that was sensitive to highly similar but unpredicted shapes (shape B vs C; see Materials and Methods for further details). First, we tested whether the LDA was able to uncover neural representations of the presented shapes, we trained and tested a shape A versus D decoder within the localizer runs in a cross-validated manner (−100 to 600 ms, relative to stimulus onset; Fig. 2*A*). These decoders were then used to test whether the predictive auditory cues induced oscillatory representations of the predicted shapes (Fig. 1*E*). Specifically, we applied the shape decoders (i.e., spatial filters) to the MEG data recorded in the interval between the predictive cues and stimulus onset (−1,250 to 0 ms relative to the onset of the first shape) in a time-resolved manner.

That is, the time-resolved data (sliding window of 28 ms, steps of 5 ms; Fig. 2*B*, Fig. S2) from the prediction interval were put through a static shape-specific spatial filter obtained on the basis of the shape localizer data, yielding a shape-specific signal for each trial every 5 ms (Fig. 2*B*). Averaging these shape-specific MEG signals over trials revealed prediction-like representations around the auditory cue offset (at ∼200 ms training and approximately −1,550 ms testing time) and closer to the onset of visual stimuli (at approximately −500 ms testing time; Fig. S2). However, these clusters did not survive correction for multiple comparisons, unlike in a previous study from our lab (Kok et al., 2017). There are several possible explanations for this discrepancy. First, in the current study, the auditory cues predicted complex shapes rather than low-level features (grating orientation), and fMRI work has shown that predicted-but-omitted gratings can be decoded from the early visual cortex, whereas prediction-but-omitted shapes cannot (Aitken et al., 2020). Second, here we used a longer cue–stimulus onset asynchrony (1,750 ms here, 750 ms in Kok et al., 2017). A longer prediction interval can (1) lead to more jitter in the onset of pre-stimulus prediction signal due to increased temporal uncertainty or (2) incentivize the brain to uses oscillations rather than sustained above baseline activity to keep predictions online, for metabolic efficiency.

**Figure 3.**
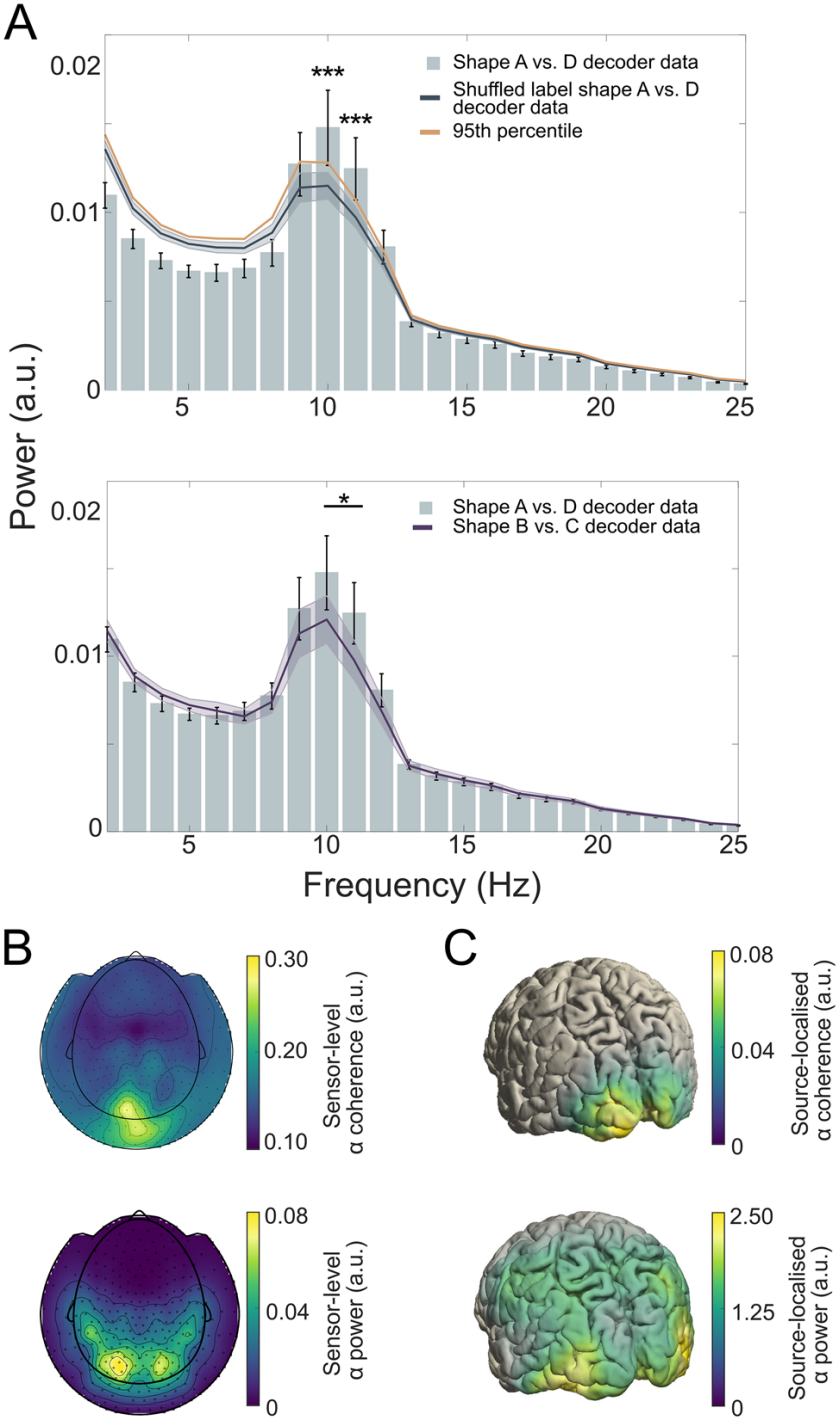
Auditory cue-induced prediction templates fluctuate at alpha frequencies. ***A***, Top, The power spectrum of pre-stimulus (−1,250 to 0 ms) shape decoding shows significant deviations from an empirical null distribution at 10 and 11 Hz (***p = 0.003). The baseline power spectrum (dark blue line) was obtained by bootstrapping (*n* = 10,000) shuffled label decoding data (*n* = 25 per participant). Mean and shaded regions indicate SD. Solid orange line indicates the 95th percentile of the null distribution. Error bars indicate SEM. ***A***, Bottom, Pre-stimulus (−1,250 to 0 ms) MEG data revealed significantly higher 10–11 Hz power for shape A versus D decoding than for shape B versus C decoding (*p < 0.05). Error bars indicate power of shape A versus D decoding; dark purple line indicates power of shape B versus C decoding (applied to identical pre-stimulus prediction data). Shaded regions and error bars indicate SEM. ***B***, Top, Topography of sensor-level alpha (10 Hz) coherence between shape-specific signals (i.e., decoder output) and pre-stimulus sensor-level raw MEG signals (−1,250 to 0 ms). ***B***, Bottom, Topography of raw pre-stimulus sensor-level alpha power (10 Hz, −1,250 to 0 ms). ***C***, Top, Source-localized alpha coherence between shape-specific and pre-stimulus raw MEG signals (−1,250 to 0 ms). ***C***, Bottom, Source-localized raw alpha power (10 Hz with 2 Hz spectral smoothing, −1,250 to 0 ms).

To test this, the shape-specific time courses were subjected to a fast Fourier transform (FFT) in order to reveal their frequency spectra (Fig. 2*B*). To establish the specificity of the neural signals induced by predictions, we created two separate baseline measurements. First, we shuffled the shape labels before training the decoder (*N* = 25 permutations per participant) to create a bootstrapped baseline (Fig. 2*C*, left, Baseline 1). Second, we trained a decoder to distinguish two shapes which were only presented in the localizer runs, but not in the prediction runs (shapes B and C). This discrimination was designed to be orthogonal to shape A versus D discrimination, which was confirmed by an absence of cross-decoding between the two decoders (Fig. S1*B,C*). The shape B versus C decoder thus provides a highly stringent baseline (Fig. 2*C*, right, Baseline 2), since it was trained to pick up neural representations of highly similar but orthogonal shapes to those that were predicted by the auditory cues. This analysis revealed that the decoded predictions oscillated at low frequencies, predominantly in the alpha frequency band (9–11 Hz; Fig. 3*A*, top). We identified significant power differences between the shape A versus D decoding data and Baseline 1, specifically at 10 and 11 Hz, exceeding the 95th percentile of the empirical null distribution (both *p* = 0.003, corrected for multiple comparisons with a FDR of 0.01). It is important to note that Baseline 1 was based on the exact same pre-stimulus data; the only difference lies in the shuffling of the shape labels for the training of the decoder. We observed increased alpha power in Baseline 1 at 10 and 11 Hz, which aligns with the generally high alpha power observed in the pre-stimulus raw MEG signal (Fig. 3*B*, bottom, 3*C* bottom). The decoder used in this analysis functions as a spatial filter, applying a linear combination of weights to all MEG sensors.

As a result, it is understandable that increased alpha power also appears in the Baseline 1 measurement. Importantly, alpha power was higher for the true shape-specific signal than for Baseline 1. This suggests that the topography of pre-stimulus alpha oscillations was more similar to the topography of the predicted shape-specific signal than expected by chance. There was also a negative difference in the power of very low frequencies (2–7 Hz) when comparing the shape A versus D decoder data to Baseline 1 data. The nature of this power difference currently is not fully understood and requires further investigation. However, these patterns have been observed in previous research using this methodology (Kerrén et al., 2018, 2022).

For further validation, we also compared the shape A versus D decoding power spectrum with the spectrum of shape B versus C decoding (Baseline 2). Based on our initial findings of significant differences in the 10 and 11 Hz frequency bins between shape A versus D decoder and Baseline 1, here we averaged over these two frequency bins (10 and 11 Hz) of the two spectra. This analysis revealed significantly higher alpha power for shape A versus D decoding than for shape B versus C decoding in the pre-stimulus window (paired one-sided *t* test, *p* = 0.0304, *t*_(31)_ = 1.9452, Fig. 3*A*, bottom). As before, it is important to note that the two spectra were based on the exact same pre-stimulus MEG data, the only difference lies in which shapes the decoders were trained to discriminate. If the pre-stimulus alpha fluctuations reflected more generic shape representations, this comparison would yield no significant differences. Therefore, the difference between these two decoding spectra demonstrates that these signals were highly specific to the shapes predicted by the auditory cues. It is important to note that the shape decoders were trained on sensory signals evoked by task-irrelevant shapes during the localizer (i.e., participants performed a fixation dimming task), ruling out contributions of explicit decision-making signals. These alpha power effects were also present in a control analysis designed to remove non-rhythmic signals, confirming the oscillatory nature of the decoded predictions (Fig. S3*A*). In sum, both analyses revealed that visual predictions induced by auditory cues led to neural representations of the predicted shapes fluctuating at an alpha rhythm prior to stimulus onset.

In order to establish a link between these shape-specific prediction signals and neural alpha oscillations, we estimated the coherence (cross-spectral density) between the two signals at the sensor level and in source space at 10 Hz (with 2 Hz spectral smoothing) between −1,250 and 0 ms.

Coherence values indicate how consistent the phase synchrony is between two time series signals. Sensor-level coherence analysis revealed high oscillatory synchrony between the shape-specific prediction signals and raw alpha oscillations, mainly over the occipital lobe (Fig. 3*B*, top), where alpha power was also the highest (Fig. 3*B*, bottom). Coherence analysis in source space using DICS (Gross et al., 2001) confirmed that the coherence between the two signals was strongest in the occipital lobe (Fig. 3*C*, top), as was raw for alpha power (Fig. 3*C*, bottom). No such coherence was present between raw alpha signals and signals obtained by training decoders using shuffled labels (i.e., Baseline 1, Fig. S4). Taken together, these results suggest that the shape-specific prediction signals revealed in the current study originate from alpha oscillations in the occipital lobe.

Note that this demonstration of predictions fluctuating with alpha oscillations crucially relied on training a decoder on a static, phase-aligned signal (obtained from the localizer). In a situation such as the current one where signals are not phase-aligned between trials (because they are modulated by endogenous oscillations), a more typical cross-validated within-experiment decoding analysis would not have succeeded, since there would not have been a consistent signal to train and test the decoder on.

### Oscillatory power of predicted shape representations modulates behavioral expectation effects

**Figure 4.**
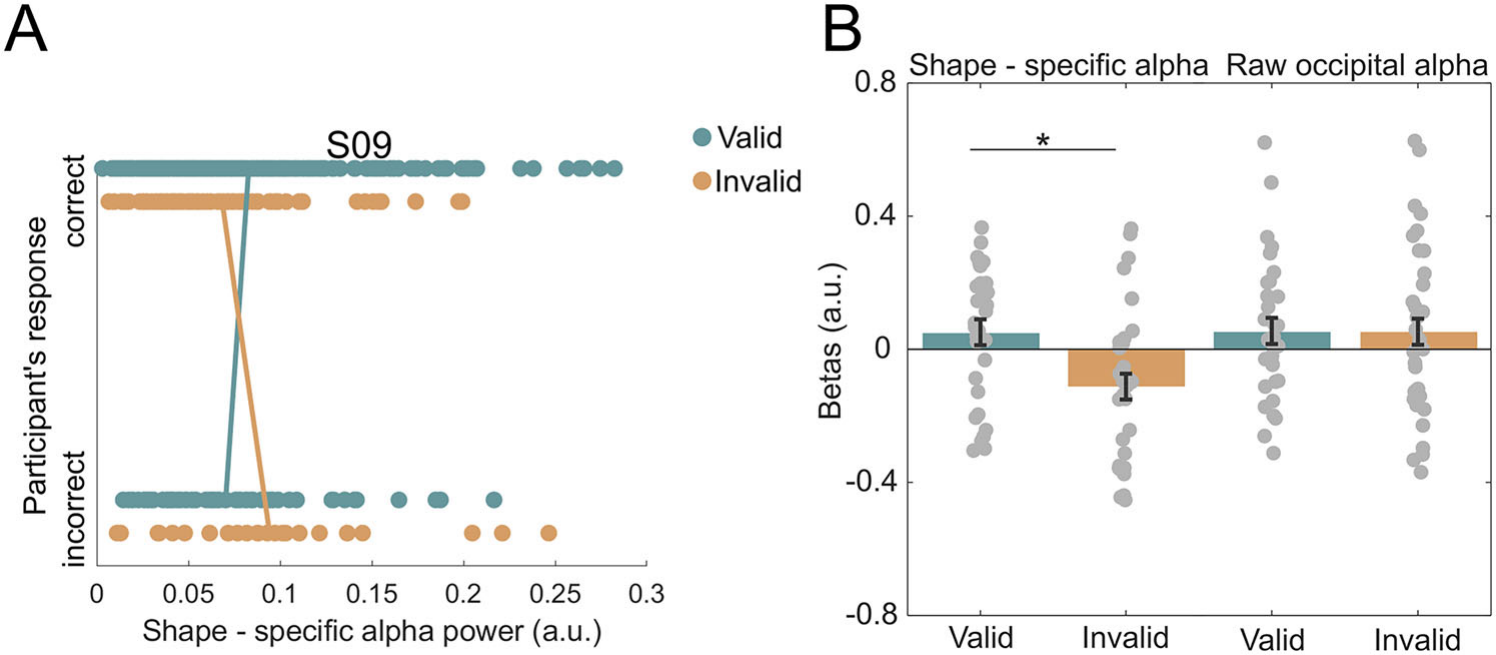
Oscillatory power of predicted shape representations modulates behavioral accuracy. ***A***, A representative participant's scatterplot of shape-specific alpha power as a function of valid versus incorrect prediction trials. We expected to observe an opposite trend between valid and invalid prediction trials, meaning for correct responses higher overall shape-specific alpha power for valid trials versus incorrect valid trials, whereas higher power was expected for incorrect invalid trials versus correct invalid trials. For all participants' scatterplots, see Figure S5. ***B***, Left, Parameter estimates (i.e., betas) of the logistic regression between the power of pre-stimulus decoding alpha (−500 to 0 ms), averaged over 8–12 Hz frequency bins, and discrimination performance, separately for valid and invalid prediction trials (*p < 0.05). ***B***, Right, Parameter estimates (i.e., betas) of the logistic regression between the power of pre-stimulus raw alpha (−500 to 0 ms), averaged over 8–12 Hz frequency bins, and discrimination performance, separately for valid and invalid prediction. No significant difference between. Dots represent individual participants; error bars indicate within-participant SEM (Cousineau, 2005; Morey, 2008).

If the strength of sensory predictions indeed modulates perceptual discrimination, there should be an opposite relationship between shape-specific pre-stimulus oscillations and behavioral performance on valid and invalid trials. To test this hypothesis, we performed a logistic regression analysis predicting behavioral accuracy from shape-specific oscillatory power in the alpha frequency range (8–12 Hz), separately on valid and invalid trials. In line with previous literature relating oscillatory power to behavioral outcome (Hanslmayr et al., 2007; Mayer et al., 2016; Samaha et al., 2018), we limited the time window of interest to −500 ms to 0 pre-stimulus, since prediction signals immediately preceding stimulus onset are most likely to impact perceptual performance (Kok et al., 2017). To assess the relationship between shape-specific decoder and raw occipital alpha, also control for nonspecific alpha effects, raw alpha power [i.e., sensor-level alpha (8–12 Hz) averaged over occipital MEG channels, for the identical time window of −500 to 0 ms] was included in the model as an additional predictor. Calculating the across-trial correlation between shape-specific alpha and raw alpha power revealed that while the two signals were correlated (mean *r* = 0.2725, *p* = 2.640 × 10^−14^, *t*_(31)_ = 12.90), they were not collinear (mean *r*^2^ < 0.1), allowing both to be included as predictors in the same regression model. This indicates a weak but consistent positive relationship between the magnitude of shape-specific and raw occipital alpha activity. The analysis revealed a significant difference between valid and invalid prediction trials, with a numerically positive relationship between pre-stimulus shape-specific alpha power and performance on valid trials and a numerically negative relationship on invalid trials (*p* = 0.014, *t*_(31)_ = 2.593; Fig. 4*B*, left). On the other hand, raw occipital alpha power did not predict behavioral outcome (Fig. 4*B*, right), reflected in a nonsignificant difference between betas of valid and invalid prediction trials (*p* = 0.934, *t*_(31)_ = 0.083). To rule out spurious effects caused by correlated regressors, we also ran the logistic regression with shape-specific alpha power as the only predictor. This control analysis reproduced the significant difference between valid and invalid trials (*p* = 0.021, *t*_(31)_ = 2.4364).

Note that the individual parameter estimates for valid and invalid trials were not significantly different from zero, while the difference between the two was. This likely reflects the fact that the individual conditions also contain unexplained, nonspecific trial-by-trial variance in behavioral performance (e.g., due to slow fluctuations in alertness) that are subtracted out in the valid versus invalid comparison. Importantly, the differential relationship between alpha power and behavior dependent on prediction validity rules out any nonspecific explanations of our results and demonstrates a strong link between neural and behavioral effects of prediction. In short, pre-stimulus content-specific alpha oscillations modulated subsequent shape discrimination accuracy, such that the difference in accuracy between validly and invalidly predicted shapes was greater when shape-specific pre-stimulus alpha power was higher.

### Stimulus predictions are driven by relatively late sensory representations

**Figure 5.**
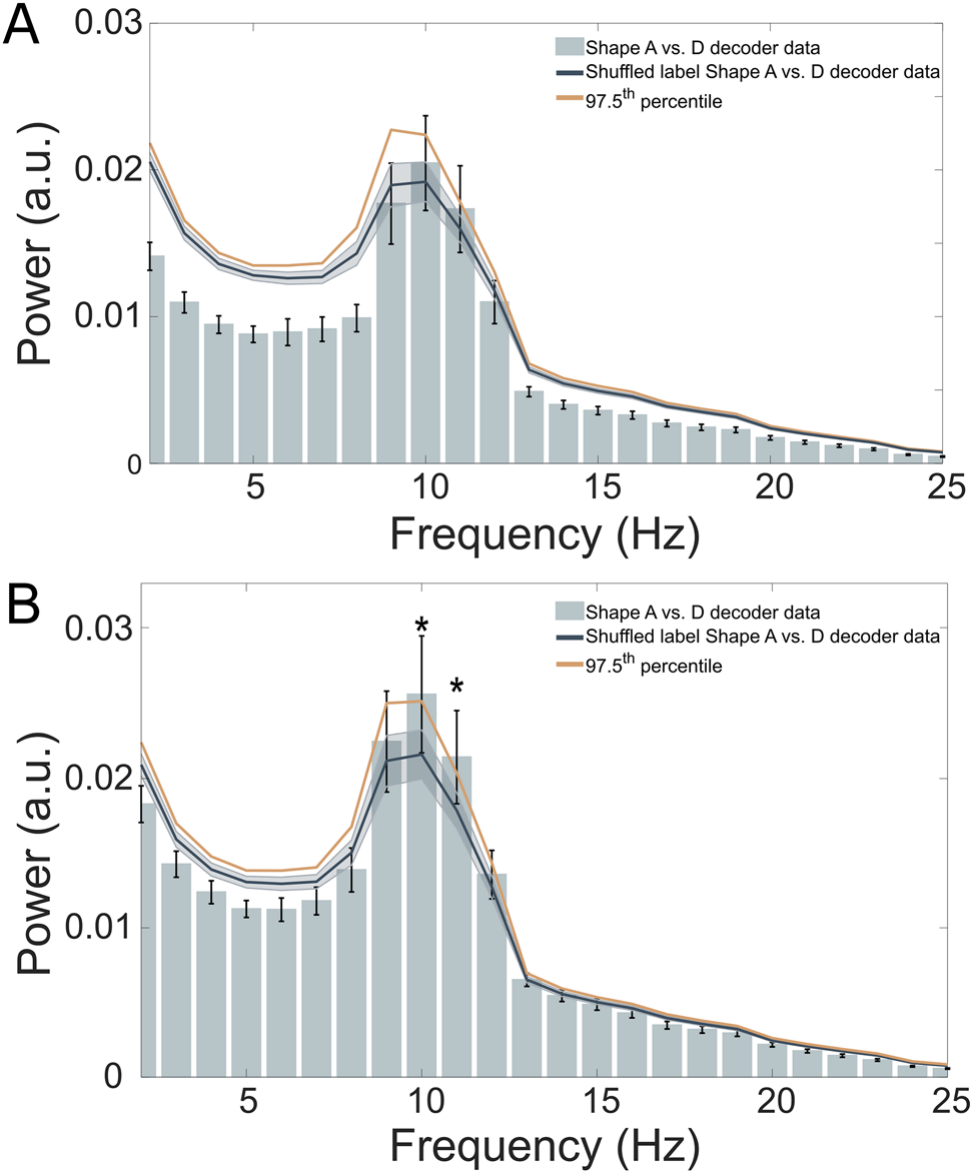
Late sensory representations drive stimulus predictions. ***A***, Power spectrum of the −1,250 to 0 ms prediction time window shape A versus D decoding, trained on the 90–120 ms post-stimulus localizer window. No significant distinctions between the shape A versus D decoder data and an empirical null distribution at 10 and 11 Hz. Mean and shaded regions indicate SD. Dark solid orange line indicates the 97.5th percentile of the null distribution, implementing a one-sided test at *p* < 0.05 while correcting for the two time windows tested here. ***B***, Power spectrum of the −1,250 to 0 ms prediction time window shape A versus D decoding, trained on the 160–190 ms post-stimulus localizer window. Statistically significant difference from an empirical null distribution at 10 and 11 Hz (*p < 0.002). The baseline power spectrum (dark blue line) was calculated as before. Mean and shaded regions indicate SD. Solid orange line indicates the 97.5th percentile of the baseline distribution.

In an exploratory analysis, we investigated whether perceptual predictions reflected early or late visual representations, by dividing the training time period (70–200 ms) into two separate windows, centered around the first (105 ms) and second (175 ms) peaks of the localizer decoding results, respectively (Fig. S1*A*). We then repeated the same frequency analysis performed on the original 70–200 ms time window for each of these separate windows. Additionally, we created distinct baseline measurements for the early and late time windows. Note that these two distinct time windows appeared to form two distinct clusters (90–120 ms and 160–190 ms) in the temporal generalization matrix of within-localizer decoding, with reduced cross-decoding between the two clusters suggesting qualitatively different representations (Fig. S1*A*). For the early training window (90–120 ms), frequency analysis of the pre-stimulus decoder time series revealed no power differences in the alpha band (10 Hz: *p* = 0.223; 11 Hz: *p* = 0.090) between the shape A versus D decoding data and an empirical null distribution (Fig. 5*A*). Furthermore, training the decoder on the later time window (160–190 ms) revealed significantly higher pre-stimulus alpha power (10 Hz: *p* = 0.016; 11 Hz: *p* = 0.002) in the shape A versus D decoding data compared with a null distribution (Fig. 5*B*, Baseline 1).

Lastly, there was a significant difference in the average power in the 10 and 11 Hz frequency bins of pre-stimulus shape A versus D decoding between the early and late training time windows (*p* = 0.005, *t*_(31)_ = −3.022). This is striking since these power spectra were calculated on the exact same MEG pre-stimulus data, the only difference was the localizer time window (90–120 ms vs 160–190 ms) on which the decoder was trained. In sum, oscillating predictions seem to reflect relatively late sensory representations (160–190 ms), rather than early feedforward sweep-like signals.

## Discussion

The present study examined the mechanisms through which predictions exert their influence on perception. Specifically, we tested whether the content of perceptual predictions was represented in oscillations and whether the power of this representation modulated performance on a visual discrimination task. To this end, we used multivariate decoding of MEG data to obtain the frequency spectrum of predicted shape representations. We revealed that predicted shape representations were strongest in the alpha frequency band (10–11 Hz; Fig. 3*A*). Source localization analyses suggest that these signals originated from the occipital lobe (Fig. 3*C*,*D*). Furthermore, we found that this stimulus-specific alpha power modulated task performance, such that higher alpha power resulted in stronger expectation effects on shape discrimination (Fig. 4*B*, left). Together, these findings suggest that sensory templates of predicted visual stimuli are represented in pre-stimulus alpha oscillations, which subsequently modulate performance on a perceptual discrimination task.

Previous studies have hypothesized that oscillations play a critical role in conveying perceptual predictions (Ergenoglu et al., 2004; VanRullen et al., 2011; Mayer et al., 2016; Auksztulewicz et al., 2017; Samaha et al., 2018). This is largely based on indirect evidence, consisting of a range of studies finding that pre-stimulus alpha oscillations modulate performance on perceptual discrimination tasks (Ergenoglu et al., 2004; Hanslmayr et al., 2007; van Dijk et al., 2008; Mathewson et al., 2009; Sherman et al., 2016). Further, there is a second body of evidence that links experimental manipulations regarding stimulus predictability to the power and phase of low-frequency oscillatory activities (Mayer et al., 2016; Sherman et al., 2016; Shen et al., 2023; Turner et al., 2023). For instance, expectations were found to rhythmically influence perceptual decision-making, where pre-stimulus occipital alpha phase modulated the strength of expectation effects on behavior (Sherman et al., 2016). Furthermore, greater representational strength was observed in alpha-band activity in cue-induced imagery (relative to self-generated). This may reflect a more precise and temporally structured encoding of predicted features, consistent with the idea that prediction signals fluctuate in the alpha rhythm, especially when externally cued and aligned to task-relevant content (Hu and Yu, 2023). Studies of selective attention have also shown that pre-stimulus alpha-band topographies contain category-specific information of to-be-attended objects, indicating that alpha activity not only reflects attentional engagement but also carries content-specific signals (Noah et al., 2020; Meyyappan et al., 2024). This also raises the important question whether expectation and attention act through the same or different neural mechanisms. Conceptually, expectation may be defined as the anticipation of likely future sensory events based on prior probabilities and attention as the selective prioritization of task-relevant stimuli or features. While some previous work suggests that they may operate independently (Kok et al., 2012a, 2016), many studies have revealed complex interactions between the two (Kok et al., 2012b; Richter and de Lange, 2019; for a review, see Schröger et al., 2015), and disentangling the two is beyond the scope of the current study.

Lastly, a recent study has demonstrated a link between pre-stimulus high alpha/low beta power and the occurrence of high confidence false percepts (Haarsma et al., 2025). However, the key hypothesis that content of perceptual predictions is embedded in pre-stimulus alpha oscillations has remained untested. In the current study, we present evidence that the representation of predicted shapes fluctuated with pre-stimulus alpha oscillations. Therefore, we speculate that pre-stimulus alpha oscillations mediate content-specific feedback signaling, meaning that stimulus content oscillates with an alpha rhythm. Our findings align with a recent study on scene imagery, which successfully decoded individual imagined scenes from alpha-band activity, demonstrating that complex visual image content can be encoded in alpha rhythms (Stecher and Kaiser, 2024). Furthermore, our findings are also in line with recent simultaneous EEG-fMRI recordings demonstrating alpha oscillations relating to feature-specific BOLD in superficial and deep layers (Clausner et al., 2024), suggesting an active involvement of alpha oscillations in stimulus processing. Together, this supports the more general proposal that oscillations can represent visual contents (Stecher et al., 2025).

While the role of pre-stimulus alpha oscillations has been extensively studied, it remains controversial. Several studies have reported that alpha-band activity is typically stronger in the absence of visual stimuli or when stimuli are unattended (Jensen and Mazaheri, 2010; Mathewson et al., 2011; Bonnefond and Jensen, 2012), leading to the influential hypothesis that alpha is predominantly an inhibitory rhythm. For example, during spatial attention tasks, alpha power shows hemispheric lateralization, with decreased alpha power contralateral to the attended location and increased power ipsilaterally (Worden et al., 2000). This pattern has been interpreted as a mechanism of top-down distractor suppression (Ferrante et al., 2023). Together, these findings have shaped the dominant view of alpha as a rhythm that plays a key role in gating information flow via inhibition.

However, our results suggest that alpha oscillations are not solely inhibitory but have an active role in cortical communication by representing the contents of feedback signals and shaping perceptual priors and stimulus templates under conditions of expectation (Bonnefond and Jensen, 2012; Samaha et al., 2018). The link between stimulus-specific pre-stimulus alpha power and expectation effects on perception revealed in the current study suggests that whether alpha facilitates or inhibits sensory processing may depend on whether inputs match or mismatch current predictions. These findings do not necessarily challenge the prevailing view of alpha as an inhibitory rhythm. Rather, they suggest that fluctuations in the predicted shape decoding time courses may reflect a distinct functional role of alpha in this context. Specifically, the magnitude of stimulus-specific alpha-band activity may represent the strength of predictive signals, with its amplitude modulating how effectively incoming sensory information is processed in the visual cortex. We believe that the prediction signals embedded in alpha oscillations in the current study reflect such stimulus-specific predictions. It is striking that these predictions modulated behavioral performance, despite the fact that the predictions were orthogonal to the task; there was a behavioral accuracy benefit when the first shape matched the prediction, and this benefit was stronger when shape-specific alpha power was higher.

While there is convergent evidence that the brain contains predictive signals (Kok et al., 2012a; Mayer et al., 2016; Aitken et al., 2020; Haarsma et al., 2023), the mechanisms through which the brain deploys these predictions remain largely unknown. Predictive coding has been suggested to involve rhythmic interactions between different frequency band activities (Arnal and Giraud, 2012; Bastos et al., 2012), where high-frequency gamma is responsible for feedforward signaling (originating predominantly from superficial layers) and alpha/beta oscillations exert top-down control (feedback predictions), emerging from deep cortical layers. Indeed, animal work investigating the frequency characteristics and cortical layer specificity of predictable information processing (Bastos et al., 2015, 2020; Chen et al., 2023) revealed that pre-stimulus alpha power is an indicator of stimulus predictability, originating from cortical layers involved in feedback signaling (Bastos et al., 2020). Our results extend these intracranial electrophysiological observations by relating pre-stimulus alpha oscillations to the contents of feedback signaling.

Our findings reveal that prediction signals manifest in a spatially specific activity pattern that fluctuates in an alpha rhythm, predominantly originating from the occipital lobe (Fig. 3*B*,*C*; Fig. S1*E*). This may be explained by neurons in the visual cortex tuned to different shapes receiving increased feedback modulation in the alpha band when their preferred shape is predicted. Alternatively, neurons tuned to the unpredicted shape may be suppressed in a rhythmic pattern, aligning with the pulse inhibition alpha theory (Klimesch et al., 2007; Mathewson et al., 2011). Future work using single-cell recordings is required to distinguish between these alternatives.

Rather than predictions being actively conveyed in an alpha rhythm, an alternative explanation of our results may be that prediction signals passively ride on ongoing alpha oscillations. Alpha oscillations are the most prominent frequency band in the awake human brain, especially in the visual cortex, and even a nonoscillatory top-down signal arriving in visual cortex may inherit these alpha rhythms. Given our finding that shape-specific alpha power has opposite effects on behavior dependent on the validity of the predictions, such a more passive explanation seems less likely. However, future research is indeed needed to properly distinguish between these hypotheses.

Exploratory analyses revealed that the oscillating prediction signals reflected relatively late sensory representations (160–190 ms localizer training window; Fig. 5*B*). We speculate that during this time period, the sensory representations captured by the decoder reflected an integration of bottom-up inputs and top-down recurrence, rather than solely the first feedforward sweep. Like the current study, previous studies have also revealed top-down modulations that reflected relatively late post-stimulus representations (i.e., 120–200 ms; Kok et al., 2017; Dijkstra et al., 2018). This may explain why predictions have been shown to modulate later sensory processing, while leaving the early feedforward sweep (<80 ms post-stimulus) mostly untouched (Alilović et al., 2019; Aitken et al., 2020).

Many prominent and influential theoretical frameworks have long speculated on the role of neural oscillations in perception (Klimesch et al., 2007; Jensen and Mazaheri, 2010; VanRullen et al., 2011). Here, we shed light on this by showing that the content of visual predictions fluctuated at alpha rhythms and these rhythmic predictions modulate subsequent perceptual performance. These findings enrich current models of perceptual inference in the human brain by revealing a possible neural mechanism through which predictions are kept online in order to guide perception.

## Data Availability

All original MEG data and all original code is deposited at OSF (https://osf.io/re6x7) and publicly available.
